# Association of whole mtDNA, an NADPH G11914A variant, and haplogroups with high physical performance in an elite military troop

**DOI:** 10.1590/1414-431X202010317

**Published:** 2021-04-26

**Authors:** C.G.M. Santos, N.G. Rolim-Filho, C.A. Domingues, M. Dornelas-Ribeiro, J.L. King, B. Budowle, R.S. Moura-Neto, R. Silva

**Affiliations:** 1 Instituto de Biologia do Exército, Rio de Janeiro, RJ, Brasil; 2 Centro de Instrução de Operações Especiais do Exército Brasileiro, Rio de Janeiro, RJ, Brasil; 3 Center for Human Identification, Department of Microbiology, Immunology, and Genetics, University of North Texas Health Science Center, Fort Worth, TX, USA; 4 Instituto de Biologia, Universidade Federal do Rio de Janeiro, Rio de Janeiro, RJ, Brasil; 5 Instituto de Biofísica Carlos Chagas Filho, Universidade Federal do Rio de Janeiro, Rio de Janeiro, RJ, Brasil

**Keywords:** Mitogenome, Physical performance, Haplogroups, Polymorphism

## Abstract

Physical performance is a multifactorial and complex trait influenced by environmental and hereditary factors. Environmental factors alone have been insufficient to characterize all outstanding phenotypes. Recent advances in genomic technologies have enabled the investigation of whole nuclear and mitochondrial genome sequences, increasing our ability to understand interindividual variability in physical performance. Our objective was to evaluate the association of mitochondrial polymorphic loci with physical performance in Brazilian elite military personnel. Eighty-eight male military personnel who participated in the Command Actions Course of the Army were selected. Total DNA was obtained from blood samples and a complete mitochondrial genome (mtDNA) was sequenced using Illumina MiSeq platform. Twenty-nine subjects completed the training program (FINISHED, ‘F'), and fifty-nine failed to complete (NOT_FINISHED, ‘NF'). The mtDNA from NF was slightly more similar to genomes from African countries frequently related to endurance level. Twenty-two distinct mtDNA haplogroups were identified corroborating the intense genetic admixture of the Brazilian population, but their distribution was similar between the two groups (F_ST_=0.0009). Of 745 polymorphisms detected in the mtDNA, the position G11914A within the NADPH gene component of the electron transport chain, was statistically different between F and NF groups (P=0.011; OR: 4.286; 95%CI: 1.198-16.719), with a higher frequency of the G allele in group F individuals). The high performance of military personnel may be mediated by performance-related genomic traits. Thus, mitochondrial genetic markers such as the *ND4* gene may play an important role on physical performance variability.

## Introduction

Human physical performance is a multifactorial and complex trait that is influenced by environmental and hereditary factors. Environmental factors alone have been insufficient to characterize all outstanding phenotypes, resulting in a growing interest in the contribution of genetic factors to performance (1–3). Recent studies involving population distribution, exercise physiology, and genetic polymorphisms have identified several loci that may potentially influence physiological features of aerobic and anaerobic performance including maximal oxygen uptake (VO_2max_), proportion of muscle fibers, muscle strength phenotypes, mitochondrial density, and energy pathways (4,5).

However, the large number of candidate genes identified and the potential polygenic basis of high performance have not been able to explain the associations between genotypes and physical performance (6). Although several polymorphisms in genes from nuclear genome have been associated with physical performance (4,7-9), associations involving isolated single nucleotide polymorphisms (SNPs) are modest (10).

Recent advances in high-performance genomic technologies have enabled the investigation of a greater number of candidate nuclear genes as well as the entire mitochondrial genome, increasing the investigative capacity of interindividual variability and its impact on physical performance and other health-related issues (11–14). The evaluation of entire sequences of mitochondrial genomes may play a prominent role in this context, especially considering that oxidative (type I) muscle fibers with high mitochondria density and high capacity for aerobic metabolism are common in endurance athletes with high VO_2max_ levels (15,16). In addition, many of the proteins involved in oxidative phosphorylation are encoded by the mitochondrial genome (mtDNA), which has led to a surge of interest in investigating mitochondrial genetic diversity and its complex interactions with the nuclear genome within the context of aerobic and anaerobic performance (2,17).

Furthermore, the human mitogenome carries information about maternal ancestry, since different mitochondrial haplogroups reflect the population density and geographical distribution of their matrilineal lineages (18). In fact, the physical performance of many elite athletes from countries that traditionally win athletic competitions has been shown to be associated with their geographical and tribal origins as well as their mitochondrial haplogroups (17,19).

Based on the above information, the study of highly heterogeneous admixed populations represents a major challenge for understanding genomic associations within and between ethnic groups. If an ethnic group has certain genetic variants differing from other groups, they can only be determined by comparing individuals from different population groups or genes from these different groups present in a mixed population rather than by comparing cases and controls from the same ethnic group (13). Thus, considering the limited genetic research within military populations, herein a study was undertaken to gain a better understanding of the genetic contribution to physical performance in Brazilian high-performance military personnel by sequencing the entire mitogenome and comparing every *loci* and haplogroup of mtDNA among two groups: one that finished the training program with another group that failed to finish.

## Material and Methods

### Subjects

Eighty-eight male military personnel who participated in the Command Actions Course (CAC) at the Special Operations Instruction Center (CIOpEsp) of the Brazilian Army volunteered for genetic analysis. Participants were assigned to one of two groups according to their level of performance: subjects who completed the course (FINISHED, ‘F'; n=29) and individuals who voluntarily left training at any time during the course and failed to complete the course (NOT FINISHED, ‘NF'; n=59). All subjects provided written informed consent. The study was approved by the Ethics Committee of Clementino Fraga Filho University Hospital (HUCFF) at the Federal University of Rio de Janeiro (CAAE/HUCFF/UFRJ, No. 67197717.0.0000.5257).

### CAC protocols from CIOpEsp

According to CAC protocol, all participants underwent a medical examination prior to testing. Participants who left the course due to lesions and illnesses, including trauma and infections were not included in the genetic study. Course activities included military physical training, orienteering, water-crossing, obstacle courses, approach marches, patrolling, and swimming. Specifically, during the first three weeks of the course, participants underwent patrol and fight training during the day and swimming during the night, followed by a couple of hours of sleep. This is a very intense course that is highly demanding physically.

The following week is the hardest phase of the course, consisting of the leadership development exercise (LDE), a 102-h continuous strenuous military field exercise. Then, patrols and typical military activities were conducted in a continuous operations setting for four cycles of 24 h, including tactical training and mission planning, an approach march (~20 km), mission performance, orienteering, water-crossing, shooting, and obstacle courses, and a return march to base (~20 km). Next, participants attended an uninterrupted six-hour combat simulation workshop. Following the LDE, participants rested for 48 h before starting another week of training. Combat stressors were progressively applied as the activities were performed.

A rated perceived exertion (RPE) scale was administered when logistically possible at 21 different moments over a 12-week period and computed only for participants who completed the course. Perceived exertion was measured using a modified Borg 1-10 Rating of Perceived Exertion scale (20) and subjects were asked to rate their levels of physical fatigue (PF) by answering the question: “In the last 24 h, what was your overall perception of physical exertion?” Perceived exertion of physical effort registers as follows: nothing at all (0); very, very light (0.5); very light (1); fairly light (2); moderate (3); somewhat hard (4); hard (5-6); very hard (7-9); and very, very hard (10). In our study, normal PF levels were considered those ranging between 0-2 on the scale. Participants maintained high levels of exertion over the 12-week period as indicated by the perceived exertion values summarized in Supplementary Figure S1.

### Blood sampling

A 4-mL aliquot of venous blood was drawn into BD Vacutainer™ K2EDTA tubes (Becton Dickinson, USA) by qualified laboratory personnel. Total DNA was extracted from 300 μL of blood using Genomic DNA Extraction kit according to the manufacturer's instructions (RBC Bioscience, Taiwan). The DNA concentration was determined using the Qubit^®^ dsDNA HS quantification kit and Qubit^®^ 2.0 fluorometer (Life Technologies, USA). The samples were normalized to 0.1 ng/µL and stored at -20°C until amplification.

### Mitogenome sequencing

Amplification of the mitochondrial genome was performed with 1 ng of total DNA and subsequently sequenced on the Illumina MiSeq platform (Illumina, USA) as described previously by our group and detailed in Supplementary Methods (21). The mitochondrial genome was indexed using the BWA tool (https://www.ncbi.nlm.nih.gov/nuccore/251831106) to generate BAM- and VCF-format files for alignment of sequences to the reference human mitochondrial genome rCRS (Access # NC_012920.1).

The aligned BAM files underwent mtDNA variant detection using Integrative Genome Viewer (IGV) software (http://software.broadinstitute.org/software/igv/). Heteroplasmy - the appearance of one position with two possible nucleotide bases - was considered with at least a 1:4 ratio of the two nucleotides in all sequences. Insertions and deletions in pyrimidine-rich regions (homopolymeric blocks) were treated as variations with no effect on the final result of the mitochondrial genome due to the potential high degree of variability in these regions within an individual. Consensus FASTA files were produced for each individual from the aligned BAM files.

### mtDNA haplogroups

The mitochondrial haplotypes were identified from analysis of VCF files using the mitoSAVE tool (22). Haplogroups were determined and checked using the following tools: Haplogrep (http://haplogrep.uibk.ac.at/index.html), mtDNA Haplogroup Analysis (http://dna.jameslick.com/mthap), and mtDNA-server (https://mtdna-server.uibk.ac.at/start.html#!run/). The simplified mtDNA lineages were determined using Mitomap Database (https://mitomap.org//pub/MITOMAP/WebHome/simple-tree-mitomap-cartoon.pdf).

Complete FASTA sequences for Africans (Supplementary Table S1) were obtained from GenBank (http://www.ncbi.nlm.nih.gov/genbank, in October 2015). The haplogroup should be specified in the description of each file and sequences should be contemporary rather than ancient in origin. Two Kenyan sequences (L0 haplogroups), nine Ethiopian sequences (three L0, four L3, and two M), and a complete Jamaican sequence (L1) were selected (Supplementary Table S1). An outgroup sequence (Neanderthal mtDNA, GenBank Accession # FM865411) was used to evaluate the quality of the tree generated.

### Statistics and data analysis

Serum cholesterol levels, height, and BMI means were compared using paired t-test. Age means were compared with Student's *t*-test. FASTA files from study participants and Africans were aligned using ClustalW tool in MEGA7 software (23). Following alignment and after a model test had been run, a maximum likelihood-based phylogenetic tree (Tamura-Nei model) was generated using 500 bootstrap replicates. Simplified mtDNA lineages from F and NF were tested by chi-squared test. The proportion of the haplogroup variance in relation to the total genetic variance represented by the FST values was tested based on Wright's F-statistics. FST values and genetic distance matrices for the SNPs and for the pairwise comparisons of the mitochondrial sequences were determined using the Arlequin v.3.5 (24). Effect sizes were obtained using Cohen's d method (80% power, α=0.05). For all tests, the significance level applied was 95%.

## Results

### Physical performance

Mean age (years) of participants was not significantly different between the groups (F: 27.7±4.2 *vs* NF: 28.6±4.0; t(86)=-1.035; P=0.304; effect size (ES) d=0.219447; 95%CI (F): 26.1-29.3), (NF)=27.6-29.7) (F + NF: 28.3±4.1; 95%CI: 27.5-29.2), indicating that passing and failing (suggesting a different physical capacity) and age were not associated with the dropout rate. The main characteristics of participants that completed the course are summarized in Supplementary Table S2.

**Figure 1:**
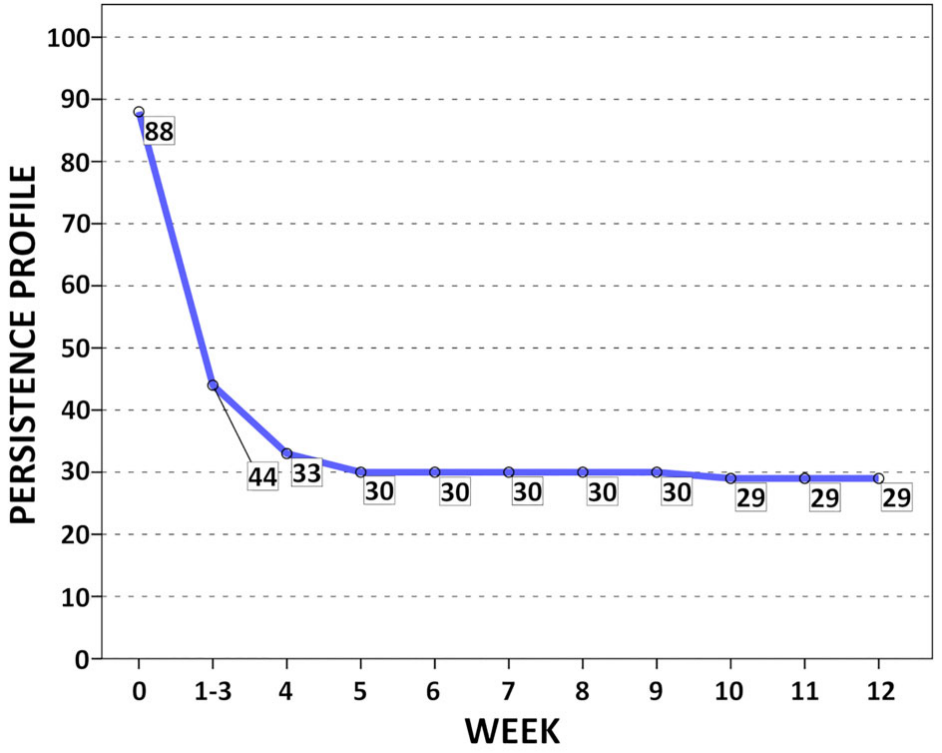
Persistence profile of the subjects who completed the course (FINISH (F) group). The numbers in boxes indicate the number of individuals who completed each week of the course.

The persistence profile of participants during the 12 weeks of the course is shown in Figure 1. The high dropout rate in the first four weeks coincided with the highest RPE and reflected the physical inability of participants in the NF group to perform vigorous activities at the beginning and during the hardest phase of the course (LDE). Results of the RPE (0-10) administered to participants are shown in Figure S1. The 29 individuals that completed the course had a mean RPE of 4.0±3.6. RPE of the NF group was not computed because most participants failed to complete the course even before undergoing the first evaluation.

### Haplogroups and simplified mitochondrial lineages

On average, 284,795 sequence reads per sample were mapped to the mitochondrial genome with read depth of 17.2×. There were 745 different polymorphic regions detected in the 88 samples. Twenty-two distinct mtDNA haplogroups were identified, indicating a high level of admixture in the Brazilian population (Supplementary Table S3). In addition, the proportion of the haplogroup variance in relation to the total genetic variance between the F and NF groups represented by the F_ST_ values was 0.0009 (PP=0.3874), indicating that haplogroup distribution was similar in the two groups (24). In addition, the distribution of simplified mtDNA lineages was not significantly different (P=0.6399) between the two groups (Supplementary Table S4).

### African mitochondrial genomes and military subjects

**Figure 2:**
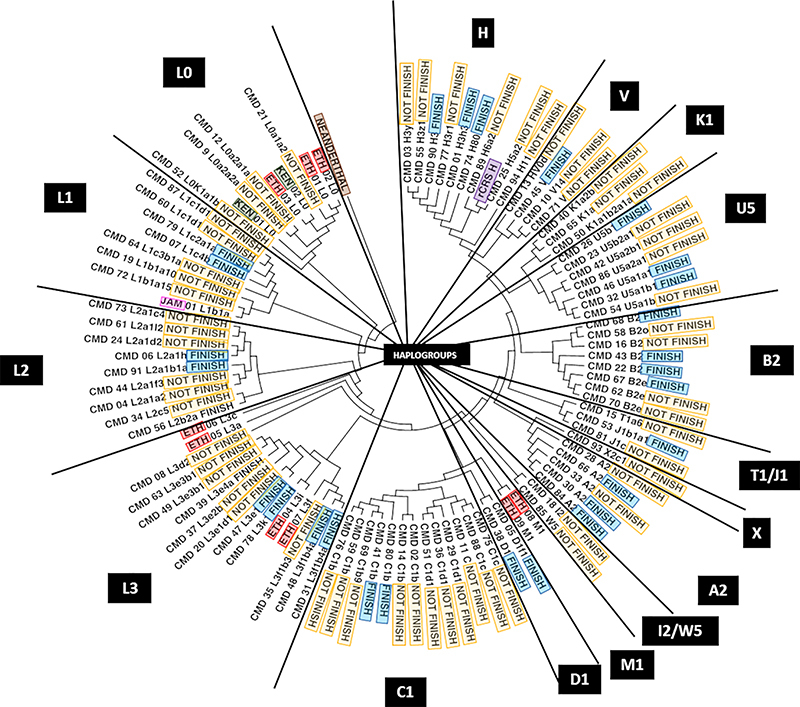
Phylogenetic tree generated by MEGA7 software with the Tamura-Nei model. In red, Ethiopian individuals (ETH) of diverse haplogroups; in gray, Kenyan individuals (KEN); in pink, a Jamaican individual (JAM); in blue, subjects who completed the course (FINISH); in yellow, who did not finish (NOT_FINISH); and in purple, the revised Cambridge Reference Sequence of mtRNA (rCRS). The squares and rectangles are the mitochondrial haplogroups presented by each group of individuals. The brown rectangle highlights the root of the tree, which represents the mitochondrial sequence of a genome outside the groups, a Neanderthal man.

Sequences from study participants were compared with those from populations or ethnicities that traditionally produce international-level elite endurance athletes to estimate the phylogenetic tree against the reference genome (Figure 2). The tree revealed a heterogeneous distribution with a clustering pattern consistent with the haplogroups previously determined for each sample. African haplogroups were grouped with similar haplogroups from Brazil. An outgroup sequence (Neanderthal mtDNA) (25) was used to evaluate the quality of the tree generated. This sequence grouped as expected, close to the oldest ancestral haplogroup (L0). Similarly, the reference genome rCRS clustered together with individuals from the same haplogroup (H). However, we found no consistent association between haplogroup distribution and physical performance.

**Figure 3:**
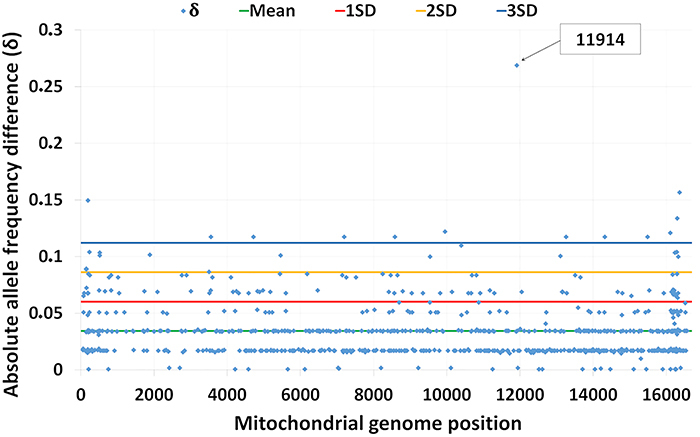
Absolute allele frequency differences (δ) between subjects who completed the course (FINISH) and those who did not (NOT_FINISH) for each polymorphic site of mitochondrial genome.

Functional analysis of F and NF sequences was performed using the absolute allele frequency difference (δ) (26) at every mitochondrial genomic position (16,569 nucleotide positions), with all positions set to the most frequent allele. Of the 745 polymorphic sites detected in F and NF sequences, only 13 had absolute allele frequency difference (δ) values greater than three standard deviations (3 SD), which are indicative of large differences between the F and NF groups (Figure 3). Of the 13 variants with δ values >3 SD, only nucleotide position G11914A exhibited a significant difference in frequency distribution between the F and NF groups (P=0.011; OR: 4.286; 95%CI: 1.198-16.719), with a higher frequency of the G allele in group F individuals (Supplementary Table S5).

## Discussion

### Course performance analysis

Physical performance studies in the military setting have unique characteristics, because military personnel often remain on-base during training, favoring the standardization of environmental aspects. In special operations forces, high-intensity training produces functional adaptations that improve performance in specific tasks. Even though they cannot be considered elite athletes, special operations troops have very high energy demands with mean expenditure values ranging from 17.1 MJ/day to 29.8 MJ/day (27–29), which is higher than that described for elite marathon runners in training (14.6 MJ/day) (30). In our study, the RPE of participants was indicative of high physical requirements. At some time-points, mean RPE in the F group was >9 (out of a maximum of 10). Due to the high requirements of the selected military, it could be speculated that individuals able to complete such a physically demanding course may have some genetic component associated with better physical performance, similar to an elite athlete or an individual belonging to a certain group of population (ethnicity/geography) (31,32).

The anthropometric phenotypes and the biochemical characteristics of the groups showed physiological changes in response to intense training (see Supplementary Table S2). The significant increase in serum HDL cholesterol levels and the reduction of LDL and body weight were used as markers of these changes. However, no reduction in BMI was detected, indicating that body fat percentage would be a better predictor of the proportions of lean mass and fat mass.

### Mitogenome analysis

We selected mitochondrial genome sequences from Kenyan and Ethiopian individuals due to the noted success of athletes from these countries in middle- and long-distance international running events, whereas the Jamaican sequence was selected because of the training of Jamaican athletes for strength-power events. L3 haplogroups have been more frequently observed among elite Kenyan athletes whereas the L0/M haplogroups are more frequent in non-athlete Kenyans. Moreover, in elite Ethiopian athletes the M haplogroup is associated with performance. Thus, selection of African sequences was centered on these haplogroups (17).

However, based on Wright's statistics (33), pairwise mtDNA comparisons showed a moderate to large difference between Africans and the NF group (F_ST_=0.12795) and the F group (F_ST_=0.18096), supporting a genetic difference for particular ethnic groups. However, F_ST_ values decreased, especially in the NF group, approaching values considered by Wright as low genetic differentiation, when comparing only individuals with typically African ‘L’ haplogroups (Supplementary Table S6). Mitochondrial sequences from the NF group were more similar to African sequences from regions/ethnic groups with a tradition of forming elite-level aerobic resistance athletes.

Despite the seemingly conflicting result, because individuals of African descent are usually associated with high aerobic endurance performance, it should be noted that the activities performed by high-intensity operational military personnel have high energy demands but slightly different characteristics. Performing strenuous activities such as carrying a heavy backpack for several kilometers and lifting heavy weights requires high aerobic resistance and high anaerobic capacity. Interestingly, different metabolic demands may influence the relative contribution of aerobic and anaerobic systems such as anaerobic glycolysis and phosphocreatine in high-performance soldiers (34,35).

### Functional mtDNA analysis

NADH dehydrogenase 4 (ND4, complex I) is a component of the electron transport chain crucial for the transport of electrons and for energy production. Located in *ND4*, the polymorphism at nucleotide position G11914A is a silent variant (i.e., synonymous) in which the most frequent nucleotide ‘G’ (codon ACG) and the less frequent ‘A’ (codon ACA) encode the same amino acid, threonine (Thr). The frequencies of the corresponding tRNAs CGT and TGT are similar in humans according to the Genomic tRNA database (GtRNAdb) (Supplementary Figure S2). However, analysis of the Codon Usage Bias (CUB) revealed that the molar ratio between the codons (ACA/ACG) in the human mitochondrial genome is 12.6 (32.7/2.6, frequency per thousand, <http://www.kazusa.or.jp/codon>, Supplementary Figure S3). CUB varies between species, among genome regions, and even within the same gene. Thus, higher codon ratios may be related to optimized fundamental cellular processes such as translation speed and fidelity, possibly resulting from selection/adaptation (36–38). It is speculated that an optimized G allele-related process may have a potential influence on physical performance. Further research on transcription efficiency of this region is warranted.

Interestingly, all L0 individuals (Kenyan, Ethiopian, and Brazilian) had nucleotide ‘A’ at position G11914A. Conversely, all individuals from haplogroup L3 (Ethiopian and Brazilian) had nucleotide ‘G’ at position G11914A. Distribution of nucleotides ‘A’ and ‘G’ varied between haplogroups L1 and L2, and only Ethiopian M haplogroups had nucleotide ‘G’ at position G11914A (Supplementary Table S7). However, no association between haplogroups and performance was found.

Sequence analysis using RNAfold tool (39) showed that the predicted secondary structures of the *ND4* mRNAs and their free energy values (genotype G: −200.52 Kcal/mol; A: −200.16 Kcal/mol) were very similar (see Supplementary Figure S4), indicating that the mRNAs have very similar stability.

**Figure 4:**
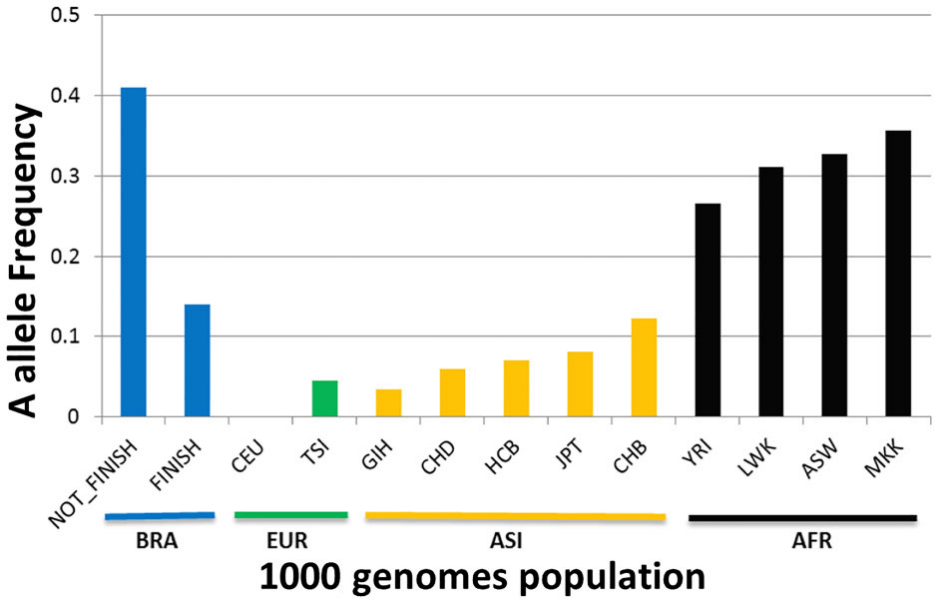
The A allele frequencies of mitochondrial variant at nucleotide position G11914A for European (EUR), Asian/Amerindian (ASI), and African populations (AFR) according to the 1000 Genomes Project database, as well as subjects who completed the course (FINISH) or did not (NOT_FINISH) in the sample from Brazil (BRA).

Analysis of mitochondrial genome data from the 1000 Genomes Project (https://www.internationalgenome.org/) revealed that the A allele at position G11914A has a heterogeneous distribution in various world populations (Figure 4). The lowest allele A frequencies were observed in populations of European origin followed by those of Asian/Amerindian origin. The highest frequencies were observed in African individuals, including two Kenyan populations (Supplementary Table S8 and Figure 4). Allele A frequencies in the NF group were similar to those of African populations and slightly lower in the F group.

For the first time, an association between mitochondrial polymorphism at position G11914A in the NADPH gene and physical performance during high-intensity exercise was detected. Mitochondrial functional assays of the variant described should provide further insight into whether this association has any causal effects.

Recently, Vellers et al. described that specific sites across the mtDNA may be related to VO_2max_ trainability (40). Therefore, we believe our findings could encourage the physical education research community to incorporate results from molecular biology and genome database studies into future research of other populations, similar to what has been done in other health science fields.

The sample size was relatively small. Because of the unique characteristics of the population, we could only analyze a small sample. In addition, due to the high complexity and intensity of the military activities performed, it was difficult to control for individual phenotypes or performance disciplines. In future studies, we intend to measure some physiological variables to better understand the physiology of high-performance groups. Besides the physical performance aspects that were preferably used in the Command Action Course, the measurement of psychological aspects could improve the understanding of performance. Lastly, greater availability of African genomes or African genomes from high-performance athletes/military personnel could greatly improve our analysis.

Military personnel and high-performance athletes may achieve remarkable results in part due to performance-related genomic characteristics. Thus, despite the lack of association with haplogroups in our investigation, mitochondrial genetic markers obtained from complete mtDNA analysis such as in *ND4* gene from athletes exposed to strong selective pressure may play an important role for understanding variability in physical performance. Genomics on targeted gene sequences and/or large-scale sequencing warrants further studies.

## Supplementary Material

Click here to view [pdf].
